# Efficacy and safety of low-dose esketamine for painless gastrointestinal endoscopy in adults: a systematic evaluation and meta-analysis

**DOI:** 10.3389/fphar.2024.1364546

**Published:** 2024-04-05

**Authors:** Juan Deng, Yun-Feng Yu, Zheng-Guo Tang, Hua-Juan Lei, Chuan-Chuan Tan

**Affiliations:** ^1^ Digestive Endoscopy Center, The First Hospital of Hunan University of Chinese Medicine, Changsha, China; ^2^ Department of Anesthesiology, The Third Hospital of Changsha, Changsha, China

**Keywords:** esketamine, propofol, anesthesia, painless gastrointestinal endoscopy, meta-analysis

## Abstract

**Object:** The benefits of low-dose esketamine for painless gastrointestinal endoscopy remain unclear. As such, the present study aimed to investigate the efficacy and safety of low-dose esketamine for this procedure.

**Methods:** Seven common databases were searched for clinical studies investigating low-dose esketamine for painless gastrointestinal endoscopy. Subsequently, a meta-analysis was performed to synthesize and analyze the data extracted from studies fulfilling the inclusion criteria.

**Results:** Meta-analysis revealed that, compared with propofol, low-dose esketamine in combination with propofol significantly reduced recovery time by 0.56 min (mean difference [MD] −0.56%, 95% confidence interval (CI) −1.08 to −0.05, *p* = 0.03), induction time by 9.84 s (MD −9.84, 95% CI −12.93 to −6.75, *p* < 0.00001), propofol dosage by 51.05 mg (MD −51.05, 95% CI −81.53 to −20.57, *p* = 0.01), and increased mean arterial pressure by 6.23 mmHg (MD 6.23, 95% CI 1.37 to 11.08, *p* = 0.01). Meanwhile, low-dose esketamine reduced injection pain by 63% (relative risk [RR] 0.37, 95% CI 0.28 to 0.49, *p* < 0.00001), involuntary movements by 40% (RR 0.60, 95% Cl 0.42 to 0.85, *p* < 0.005), choking by 42% (RR 0.58, 95% Cl 0.38 to 0.88, *p* = 0.01), bradycardia by 68% (RR 0.32, 95% Cl 0.18 to 0.58, *p* = 0.0002), hypotension by 71% (RR 0.29, 95% Cl 0.21 to 0.40, *p* < 0.00001), respiratory depression by 63% (RR 0.37, 95% 0.26 to 0.51, *p* < 0.00001), additional cases of propofol by 53% (RR 0.47, 95% Cl 0.29 to 0.77, *p* = 0.002), and increased hypertension by 1000% (RR 11.00, 95% Cl 1.45 to 83.28, *p* = 0.02). There were no significant differences in mean heart rate, mean oximetry saturation, delirium, dizziness, vomiting, tachycardia, and hypoxemia. Subgroup analyses revealed that, compared with other dose groups, 0.25 mg/kg esketamine afforded additional benefits in recovery and induction time, mean arterial pressure, involuntary movements, hypoxemia, and respiratory depression.

**Conclusion:** Low-dose esketamine was found to be safe and effective for providing anesthesia during gastrointestinal endoscopy, with 0.25 mg/kg identified as the optimal dose within the dosage ranges examined. However, caution should be exercised when administering this drug to patients with inadequate preoperative blood pressure control.

## 1 Introduction

As a commonly used method, gastrointestinal endoscopy is considered to be the gold standard for the clinical diagnosis of gastrointestinal diseases (Wallace et al., 2017). However, general endoscopy not only causes irritant responses, such as nausea, vomiting, and choking (Zheng et al., 2022), but also triggers autonomic nervous responses, including sweating, bradycardia, dizziness, and hypotension (Eberl et al., 2012). These adverse reactions increase patient anxiety and affect endoscopic outcomes (Qt et al., 2016). The use of sedation during gastrointestinal endoscopy can relieve the associated physical and psychological stress and improve the outcome of endoscopic procedures (Early et al., 2018). Propofol, a sedative with the advantages of rapid onset, rapid recovery, and complete metabolism, has been widely used in painless gastrointestinal endoscopy (Zhang et al., 2018; Hao et al., 2020). However, propofol does not have an analgesic effect (Vargo et al., 2012), and when used alone for painless gastrointestinal endoscopy, is prone to possible adverse reactions such as mental excitement, respiratory depression, hemodynamic instability, and cardiac arrest (Marik, 2004). Ketamine is considered to be a drug that can assist in propofol anesthesia. Relevant studies have shown that low-dose ketamine combined with propofol anesthesia can provide stable respiratory and circulatory status and reduce the initial effective dose and dosage of propofol (Eberl et al., 2020; Song et al., 2022). However, ketamine may also induce adverse reactions, such as increased intracranial pressure, dizziness, vomiting, and hallucinations, which limit its widespread use (Craven, 2007). Therefore, the search for new anesthesia-assisted drugs is imperative to reduce the potential risks associated with propofol use.

Esketamine, a ketamine isomer, is a novel N-methyl-D-aspartic acid (NMDA) receptor antagonist. It has pharmacological properties similar to ketamine but has twice the affinity for NMDA receptors and twice the sedative and analgesic effects of ketamine. However, the Food and Drug Administration (FDA) has only approved esketamine for the treatment of depression and not for anesthesia. In Europe and China, the European Medicines Agency (EMA) and the National Medical Products Administration (NMPA) have approved esketamine for general anesthesia as well as perioperative sedation and analgesia, respectively. Esketamine is generally used as an assistive drug in combination with other local or general anesthetics for minor surgeries or diagnostics that do not require muscle relaxation (Wang et al., 2019). Studies have shown that esketamine is associated with a lower rate of adverse reactions than ketamine and demonstrates strong potential to assist anesthesia (Wang et al., 2019). Low-dose esketamine is defined as a dosage of esketamine less than or equal to 0.25 mg/kg (Wei et al., 2023). Previous studies have shown that low-dose esketamine has sympathomimetic effects (Timm et al., 2008). When it combined with propofol, the suppression of circulation and respiration was significantly lower than that with other analgesics combined with propofol (Shah et al., 2011; Yilmaz et al., 2020). However, the efficacy and safety of low-dose esketamine for painless gastrointestinal endoscopy remain controversial. As such, the present study aimed to evaluate the effectiveness and safety of low-dose esketamine for painless gastrointestinal endoscopy through meta-analysis, with the aim of providing a reference for the development of anesthesia methods for painless gastrointestinal endoscopy in adults.

## 2 Methodology

The present study was performed in accordance with the Preferred Reporting Items for Systematic Reviews and Meta-analyses (PRISMA) (Crowther et al., 2010).

### 2.1 Literature search

A literature search of 4 English databases (Embase, PubMed, the Cochrane Library, Web of Science) and 3 Chinese databases (China National Knowledge Infrastructure [CNKI], Vip, and Wanfang) for studies investigating low-dose esketamine for painless gastrointestinal endoscopy, published up to October 2023, was performed. The medical subject terms used for the search were “Esketamine and Endoscopes,” and free text terms were obtained from the MeSH and Vip databases. Medical subject and free-text terms were combined for the search.

### 2.2 Inclusion and exclusion criteria

Studies fulfilling the following inclusion criteria were selected: study subjects were patients undergoing painless gastrointestinal endoscopy; propofol was administered to the control group and low-dose esketamine (≤0.25 mg/kg) combined with propofol was administered to the experimental group; the time endpoints (recovery and induction time), vital signs (mean arterial pressure [MAP], mean heart rate, mean oxygen saturation), adverse events (injection pain, involuntary movements, delirium, dizziness, choking, vomiting, bradycardia, tachycardia, hypotension, hypertension, hypoxemia, respiratory depression), and propofol use (propofol dosage, number of additional cases of propofol) were reported; and randomized controlled trials (RCTs).

Studies with duplicate data, those with incomplete, missing, or unavailable data, and those that included subjects <18 years of age were excluded.

### 2.3 Literature screening, data extraction, and assessment of risk of bias

First, all studies retrieved in the literature search were imported into Endnote (Clarivate, London, United Kingdom). Duplicates were removed, and the remaining studies were screened layer-by-layer according to the inclusion and exclusion criteria. Second, the included studies were classified and sorted, and baseline information of each study was entered into a basic characteristics table, including author, year of publication, sample size, intervention, examination type, gender, mean age, body mass index (BMI), and American Society of Anesthesiologists (ASA) classification. Finally, risk of bias was assessed using the Cochrane Risk-of-Bias tool. All tasks were performed independently by 2 of the authors (JD and YY) and any disagreements were adjudicated by a third author (CT).

### 2.4 Statistical analysis

Meta-analysis was performed using Review Manager version 5.3 (Copenhagen: Nordic Cochrane Centre, The Cochrane Collaboration). The relative risk (RR) and 95% confidence interval (CI) were used as effect sizes for dichotomous variables, and the mean difference (MD) and corresponding 95% CI were used as effect sizes for continuous variables. Heterogeneity was assessed according to the I-squared (I^2^) statistic. When I^2^ < 50%, the fixed effects model was used for analysis; otherwise, the random effects model was used. Publication bias was assessed by performing Egger’s test using Stata version 15.0 (StataCorp LLP, College Station, TX, United States); *p* > 0.1 suggested the absence of publication bias.

## 3 Results

### 3.1 Literature search and screening

**FIGURE 1:**
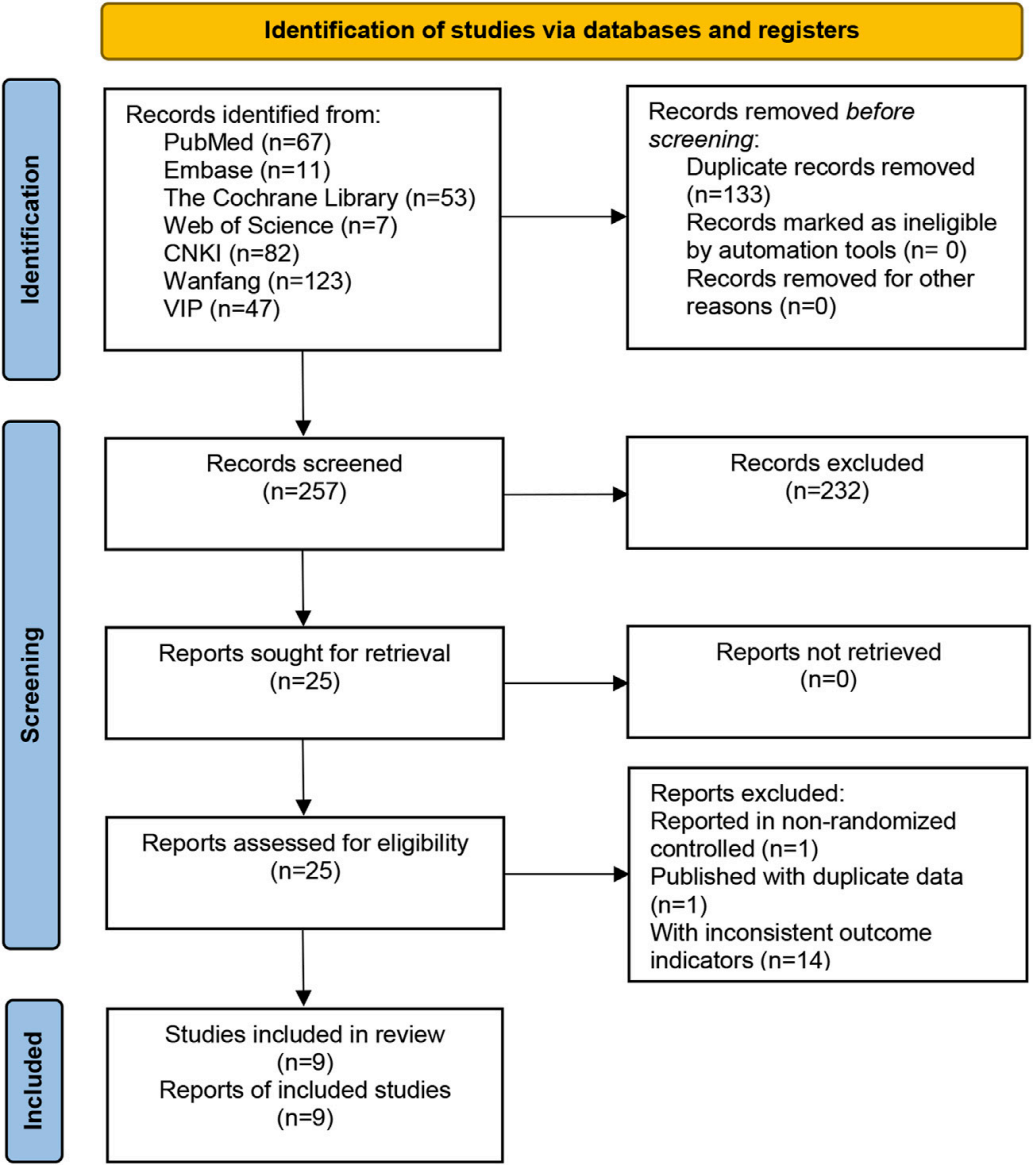
Literature screening flowchart.

A total of 390 relevant studies were retrieved from the 7 databases, and 9 relevant studies were included after layer-by-layer screening. A flow-diagram illustrating the study selection process in presented in Figure 1.

### 3.2 Basic characteristics of the included studies

Nine clinical studies were included, with study centers located in China, and a total sample size of 1144 subjects, among whom 481 underwent propofol anesthesia and 663 underwent low-dose esketamine and propofol anesthesia (Table 1).

**TABLE 1:** Basic characteristics of the included studies.

Author name	Sample size	Patient number	Intervention	Examination type	Male (%)	Age (years)	BMI (kg·m-2)	ASAI (%)
Liu et al. (2023)	76	38	0.2 mg/kg Esketamine 1 mg/kg Propofol	Gastroscopy	42.1	45.7	21.57	73.7
		38	0.9% Nacl and 1 mg/kg Propofol	Gastroscopy	31.5	49.0	22.4	73.7
Zhan et al. (2022)	260	65	0.05 mg/kg Esketamine 1.5 mg/kg Propofol	Gastrointestinal endoscopy	41.5	42.7	22.74	60.0
		65	0.1 mg/kg Esketamine 1.5 mg/kg Propofol	Gastrointestinal endoscopy	50.8	45.9	23.06	64.6
		65	0.2 mg/kg Esketamine 1.5 mg/kg Propofol	Gastrointestinal endoscopy	53.8	44.4	21.99	64.6
		65	0.9% Nacl and 1.5 mg/kg Propofol	Gastrointestinal endoscopy	41.5	44.9	22.67	66.2
Zheng et al. (2023)	104	52	0.25 mg/kg Esketamine 2 mg/kg Propofol	Gastroscopy	61.5	42.2	31.7	23.1
		52	0.9% Nacl and 2 mg/kg Propofol	Gastroscopy	65.4	41.1	31.4	21.2
Chen et al. (2022)	102	51	0.25 mg/kg Esketamine 2 mg/kg Propofol	Gastrointestinal endoscopy	52.9	45.0	23.01	51.0
		51	2 mg/kg Propofol	Gastrointestinal endoscopy	56.9	45.7	22.7	54.9
Lu et al. (2023)	172	87	0.9% Nacl and 1.5–2.5 mg/kg Propofol	Gastrointestinal endoscopy	55.2	51.0	23.4	—
		85	0.2 mg/kg Esketamine 1.5–2.5 mg/kg Propofol	Gastrointestinal endoscopy	60.0	51.4	23.8	—
Sheng et al. (2022)	60	30	0.2 mg/kg ESketamine 1–2 mg/kg Propofol	Gastrointestinal endoscopy	50.0	45.2	23.9	60.0
		30	0.9% Nacl and 1–2 mg/kg Propofol	Gastrointestinal endoscopy	66.7	43.7	22.3	60.0
Wan et al. (2022)	100	50	0.25 mg/kg Esketamine 2.0–2.5 mg/kg Propofol	Gastrointestinal endoscopy	58.0	52.4	23.9	36.0
		50	2.0–2.5 mg/kg Propofol	Gastrointestinal endoscopy	48.0	51.5	24.3	40.0
Wang et al. (2023)	150	50	0.15 mg/kg Esketamine 1.0–3.5 mg/kg Propofol	Gastrointestinal endoscopy	48.0	51.2	23.5	30.0
		50	0.25 mg/kg Esketamine 1.0–3.5 mg/kg Propofol	Gastrointestinal endoscopy	50.0	49.4	24.0	32.0
		50	0.9% Nacl and 1.0–3.5 mg/kg Propofol	Gastrointestinal endoscopy	46.0	49.1	22.9	34.0
Zhao et al. (2023)	120	60	0.25 mg/kg Esketamine 2.0–2.5 mg/kg Propofol	Gastrointestinal endoscopy	66.7	68.0	22.4	46.7
		60	2.0–2.5 mg/kg Propofol	Gastrointestinal endoscopy	71.7	70.0	22.6	41.7

### 3.3 Risk of bias assessment

**FIGURE 2:**
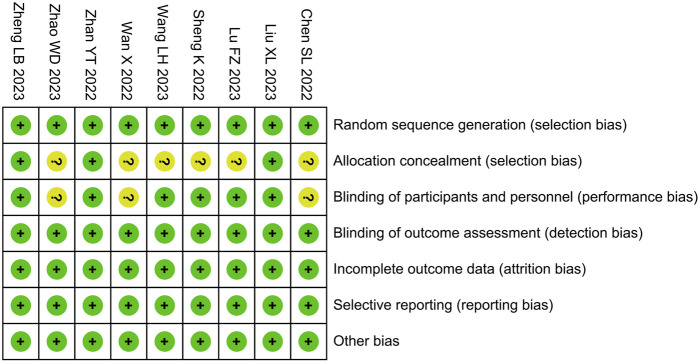
Risk of bias assessment.

Among the included studies, risk of bias in allocation concealment and intervention blinding was unclear in ;6 and 3 studies, respectively; the risk of bias in other areas was low (Figure 2).

### 3.4 Meta-analysis

#### 3.4.1 Time endpoints

**FIGURE 3:**
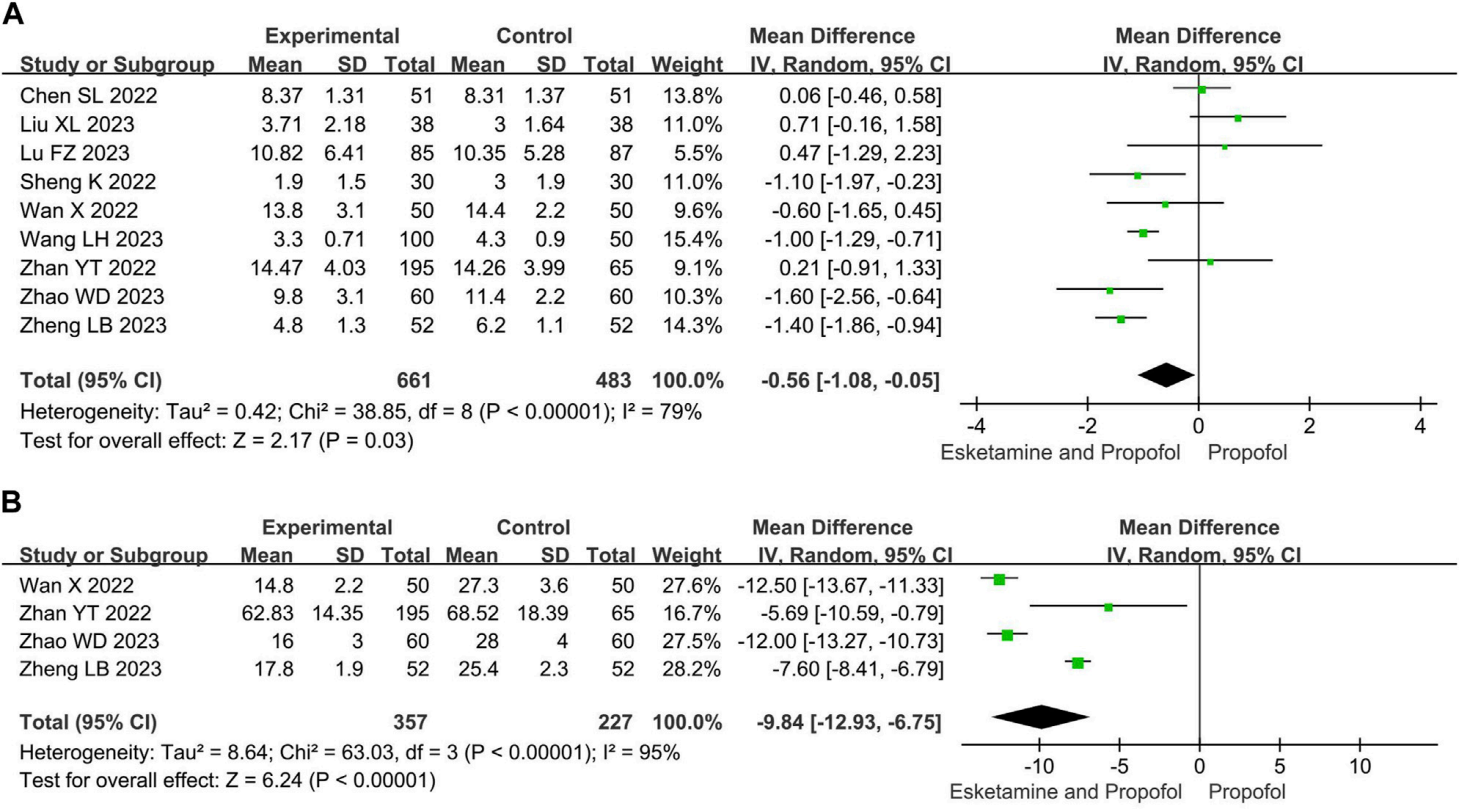
Meta-analysis results for time endpoints of esketamine and propofol compared to propofol. **(A)** Recovery time, **(B)** Induction time. The green squares represent the mean difference for each study.

Compared with the propofol, the combination of esketamine with propofol significantly reduced recovery time by 0.56 min (MD −0.56, 95% CI −1.08 to −0.05, *p* = 0.03) and induction time by 9.84 s (MD −9.84, 95% CI −12.93 to −6.75, *p* < 0.00001) (Figure 3).

#### 3.4.2 Vital signs

**FIGURE 4:**
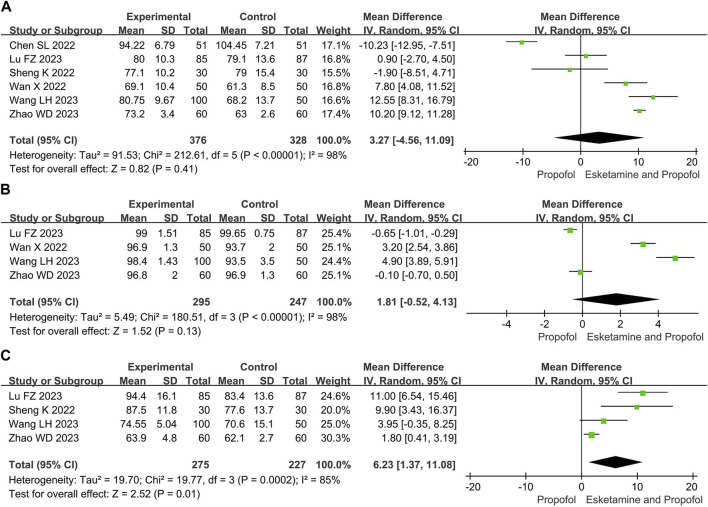
Meta-analysis results for vital signs of esketamine and propofol compared to propofol. **(A)** Mean heart rate, **(B)** Mean oxygen saturation, **(C)** Mean arterial pressure. The green squares represent the mean difference for each study.

Compared with the propofol, the combination of esketamine with propofol significantly increased MAP by 6.23 mmHg (MD 6.23, 95% CI 1.37 to 11.08, *p* = 0.01), whereas there were no significant differences in mean heart rate (MD 3.27, 95% Cl −4.56 to 11.09, *p* = 0.41) and mean oxygen saturation (MD 1.81, 95% Cl −0.52 to 4.13, *p* = 0.13) (Figure 4).

#### 3.4.3 Adverse events

Compared with the propofol, the combination of esketamine with propofol significantly reduced injection pain by 63% (RR 0.37, 95% Cl 0.28 to 0.49, *p* < 0.00001), involuntary movements by 40% (RR 0.60, 95% Cl 0.42 to 0.85, *p* = 0.005), choking by 42% (RR 0.58, 95% Cl 0.38 to 0.88, *p* = 0.01), bradycardia by 68% (RR 0.32, 95% Cl 0.18 to 0.58, *p* = 0.0002), hypotension by 71% (RR 0.29, 95% CI 0.21 to 0.40, *p* < 0.00001), respiratory depression by 63% (RR 0.37, 95% Cl 0.26 to 0.51, *p* < 0.00001), and increased hypertension by 1000% (RR 11, 95% Cl 1.45 to 83.28, *p* = 0.02), There were no statistical differences in delirium, dizziness, vomiting, tachycardia, and hypoxia between the groups (Table 2).

**TABLE 2:** Meta-analysis results for adverse events.

Outcome	Experimental (events/total)	Control (events/total)	I^2^	RR (95%CI)	*p*-Value
Injection pain	57/495	119/315	22	0.37 (0.28, 0.49)	<0.00001
Involuntary movement	209/542	185/372	80	0.60 (0.42, 0.85)	0.005
Delirium	0/336	1/206	—	0.33 (0.01, 8.00)	0.50
Dizziness	68/571	37/393	0	1.23 (0.87, 1.74)	0.23
Choking	138/431	102/303	66	0.57 (0.36, 0.91)	0.02
Vomiting	10/530	13/352	18	0.61 (0.28, 1.31)	0.20
Bradycardia	12/365	33/317	0	0.32 (0.18, 0.58)	0.0002
Tachycardia	34/138	8/88	73	2.98 (0.72, 12.37)	0.13
Hypotension	46/611	115/433	44	0.29 (0.21, 0.40)	<0.00001
Hypertension	10/103	0/103	0	11 (1.45, 83.28)	0.02
Hypoxemia	84/521	66/343	64	0.62 (0.33, 1.15)	0.13
Respiratory depression	32/292	85/242	0	0.37 (0.26,0.51)	<0.00001

#### 3.4.4 Propofol use

**FIGURE 5:**
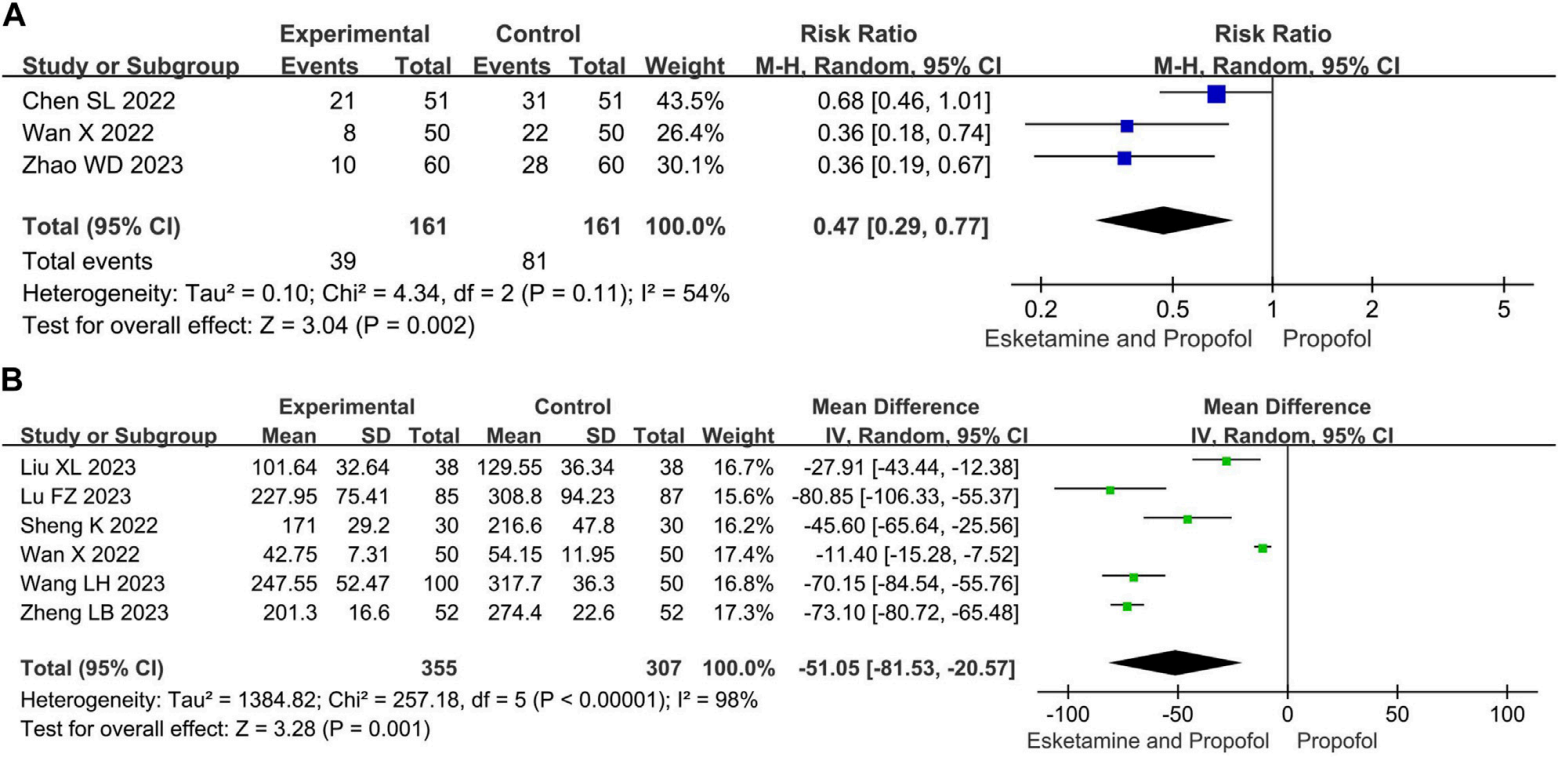
Meta-analysis results for the propofol use of esketamine and propofol compared to propofol. **(A)** Number of additional doses of propofol, **(B)** Propofol dosage. The green squares represent the mean difference for each study, while the blue squares represent the risk ratio for each study.

Compared with the propofol, the combination of esketamine with propofol significantly reduced propofol dosage by 51.05 mg (MD −51.05, 95% CI −81.53 to −20.57, *p* = 0.01) and the number of additional doses of propofol by 53% (RR 0.47, 95% Cl 0.29 to 0.77, *p* = 0.002) (Figure 5).

### 3.5 Subgroup analysis

**FIGURE 6:**
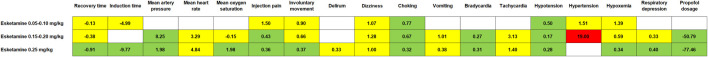
Subgroup analysis results for different doses of esketamine and propofol compared to propofol. The white grid indicates that no relevant data are available. The yellow grid indicates no significant difference. The green grid indicates that there is a significant difference and that the difference is beneficial. The red grid indicates that there is a significant difference, but the difference is harmful.

Subgroup analysis divided esketamine into 0.05–0.10 mg/kg, 0.15–0.20 mg/kg, and 0.25 mg/kg dosage groups to investigate the effect of dosage on the anesthetic effect. Results of analysis revealed that, compared with the control group, the combination group of esketamine 0.05–0.10 mg/kg significantly reduced choking and hypotension, while other indicators were equivalent. The combination group of esketamine 0.15–0.20 mg/kg significantly reduced injection pain, choking, bradycardia, hypotension, propofol dosage, and increased MAP and hypertension, while other indicators were equivalent. The combination group of esketamine 0.25 mg/kg significantly reduced recovery and induction time, MAP, mean oxygen saturation, injection pain, involuntary movements, choking, bradycardia, hypotension, hypoxia, respiratory depression, and propofol dosage, while other indicators were equivalent (Figure 6).

### 3.6 Publication bias assessment

**FIGURE 7:**
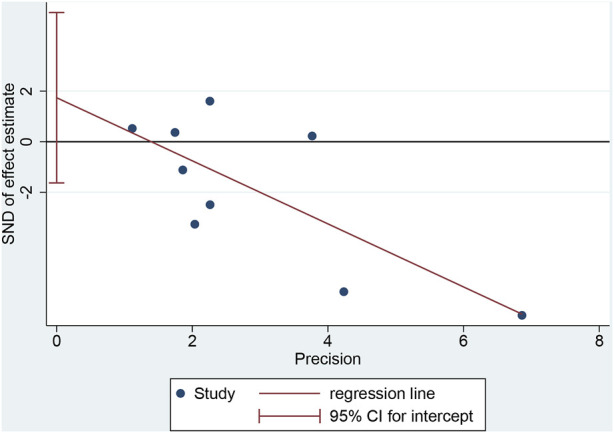
Assessment of publication bias.

Egger’s test for recovery time yielded a *p*-value of 0.262, suggesting no significant publication bias (Figure 7).

## 4 Discussion

### 4.1 Background and significance of the study

The American Society of Gastrointestinal Endoscopy (ASGE) recommends sedation during gastrointestinal endoscopy to reduce patient discomfort and improve examination efficiency (Early et al., 2018). It has been reported that the sedation rate for gastrointestinal endoscopy in the United States is >95% (Cohen et al., 2006). With the development of anesthesia technology, painless gastrointestinal endoscopy has become increasingly important. Esketamine is a psychiatric drug mainly used to treat depression in the United States. The NMPA approved esketamine for the market in 2019, and it is used for the treatment of depression, perioperative sedation, and analgesia in China. A previous meta-analysis reported that subclinical doses of esketamine under non-intubated general anesthesia reduced recovery time, injection pain, involuntary movements, choking, bradycardia, hypotension, and propofol dosage, and increased MAP (Chen et al., 2023). However, there have been no meta-analyses investigating low-dose esketamine for painless gastrointestinal endoscopy; therefore, its specific risks and benefits are unclear. To the best of our knowledge, this is the first meta-analysis to investigate low-dose esketamine for painless gastrointestinal endoscopy to explore its utility in assisting anesthesia in gastrointestinal endoscopy.

### 4.2 Evaluation of effectiveness and safety

Recovery time, induction time, injection pain, involuntary movements, delirium, dizziness, choking, and vomiting are indicators reflecting the status of the nervous system. Meta-analysis revealed that, compared with the control group, the esketamine combination group had significantly reduced recovery time, induction time, injection pain, involuntary movements, and choking, whereas there were no significant differences in delirium, dizziness, and vomiting. A meta-analysis by Chen et al. (Chen et al., 2023) described the effects of subclinical esketamine doses under non-intubated general anesthesia. Although that study supports our results with regard to injection pain, involuntary movements, delirium, and vomiting, it did not analyze the effect of esketamine on induction time. Compared to previous meta-analyses, our meta-analysis found an additional benefit of reduced recovery time and induction time with low-dose esketamine, suggesting that low-dose esketamine can reduce neurological-related risks in anesthesia for gastrointestinal endoscopy.

MAP, mean heart rate, bradycardia, tachycardia, hypotension, and hypertension are among indicators reflecting the status of the cardiovascular system. Meta-analysis revealed that, compared with the control group, the esketamine combination group had significantly increased MAP and the incidence of hypertension and reduced the incidence of bradycardia and hypotension, whereas there were no significant differences in mean heart rate and the incidence of tachycardia. This suggests that low-dose esketamine is able to attenuate depression of the cardiovascular system caused by propofol, implying that it may be beneficial in patients with concomitant morbid sinus node syndrome or hypothyroidism. Similarly, a meta-analysis by Chen et al., 2023 supported the role of subclinical doses of esketamine in improving MAP and blood pressure in patients undergoing surgery under non-intubated general anesthesia. However, we found that low-dose esketamine increased the risk for hypertension, which may occur primarily in patients with poor preoperative blood pressure control. Therefore, controlling blood pressure below target levels before painless gastrointestinal endoscopy is necessary, and we recommend that esketamine be used with caution in patients with unsatisfactory preoperative blood pressure control. Interestingly, our study demonstrated that esketamine reduced the incidence of bradycardia without significantly affecting mean heart rate. This contradictory result may stem from statistical heterogeneity. When we performed the analysis using a fixed effects model, the effect of esketamine on mean heart rate demonstrated a significant difference (MD 7.07, 95% CI 6.17 to 7.98, *p* < 0.00001).

Mean oxygen saturation, hypoxemia, and respiratory depression are indicators of the respiratory system. Meta-analysis revealed that compared with the control group, the esketamine combination group exhibited significantly reduced respiratory depression, whereas there were no significant differences in the incidence of mean oxygen saturation and hypoxemia. This suggests that low-dose esketamine attenuated the respiratory depressive effects of propofol. A meta-analysis by Chen et al., 2023 revealed that subclinical doses of esketamine reduced the incidence of apnea and intraoperative asphyxia, supporting our hypothesis. However, it did not analyze the effect of esketamine on mean oxygen saturation. Huang et al., 2023 reported that esketamine had no effect on mean oxygen saturation, which is consistent with our results. It is worth noting that Feng et al., 2022 found that esketamine significantly reduced the risk for hypoxia in elderly patients, which may be attributed to the fact that alveolar surface area, lung compliance, and respiratory center sensitivity to hypoxia and hypercapnia are lower than those in average adults, making propofol-induced hypoxemia more common in the elderly. At this point, The effect of esketamine on reducing propofol-induced respiratory depression was more significant.

Regarding the use of propofol, compared with the control group, the esketamine combination group had a reduced propofol dosage and number of additional cases of propofol. This suggests that less propofol is needed to achieve the same level of sedation when combined with esketamine, which can facilitate postoperative recovery. Related studies have shown that routine induction doses of propofol can lead to a 25%–40% decrease in basal blood pressure and respiratory depression in 25%–30% of subjects (Zhao and Li, 2021). It has also been reported that >50% of older patients who undergo painless outpatient gastrointestinal endoscopy exhibit postoperative cognitive dysfunction, which may increase the risk for developing Alzheimer’s disease (Yao and Li, 2010). Adverse events associated with propofol are closely related to dose, and reducing propofol dosage may be an important mechanism for achieving the additional benefits of low-dose esketamine in assisted anesthesia.

In summary, this study found that low-dose esketamine significantly increased mean arterial pressure, reduced propofol dosage, injection pain, bradycardia, and hypotension, while had no significant impact on delirium, dizziness, or vomiting, which is consistent with the meta-analysis results by Chen et al. Differently, we identified additional benefits of low-dose esketamine in reducing recovery time, induction time, choking, and respiratory depression in patients undergoing painless gastrointestinal endoscopy, as well as potential risks in increasing hypertension. It supported the use of low-dose esketamine for improved neurological and respiratory safety during painless gastrointestinal endoscopy.

### 4.3 Analysis of treatment mechanisms

Esketamine is an isomer of ketamine, and is a high-affinity, noncompetitive inhibitor of the NMDA receptor. It has the same site of action and pharmacological mechanism as ketamine (Yang et al., 2023), but has twice the affinity for NMDA receptors (Zanos et al., 2018). This characteristic grants esketamine with the ability to exert central inhibitory effects, as well as sympathomimetic and respiratory center stimulation effects. These mechanisms may be responsible for the beneficial effects of esketamine in anesthesia. First, esketamine exerts central inhibitory effects, which may reduce induction time and involuntary movements by noncompetitively antagonizing NMDA receptor-mediated glutamate entry into the GABAergic nervous system (Liu et al., 2023). Blockade of NMDA receptors by esketamine can induce sedation and promote analgesia, and can also synergize with propofol to reduce propofol dosage, favorably reducing choking associated with endoscope placement and shortening recovery time. However, excessive NMDA receptor blockade can cause psychotomimetic reactions such as delirium and dizziness (Annetta et al., 2005). Therefore, low-dose esketamine may improve the safety of clinical use. Second, esketamine exhibits sympathomimetic effects (Eberl et al., 2020). It can increase MAP and mean heart rate and reduce the occurrence of bradycardia and hypotension by promoting the release of catecholamines and inhibiting the reuptake of norepinephrine. Third, esketamine has a stimulatory effect on the respiratory center. It stimulates the respiratory center by blocking NMDA receptors and increasing carbon dioxide sensitivity, thereby reducing the incidence of respiratory depression (Jonkman et al., 2018).

### 4.4 Subgroup analysis

Subgroup analyses revealed that esketamine 0.05–0.10 mg/kg only reduced the incidence of choking and hypotension. Esketamine 0.15–0.20 mg/kg afforded benefits only for MAP, injection pain, choking, bradycardia, and hypotension. Compared with the overall analysis of low-dose esketamine, esketamine 0.05–0.10 mg/kg and 0.15–0.20 mg/kg did not significantly reduce recovery time, induction time, involuntary movements, and respiratory depression. We speculated that the observed benefits in overall analysis, such as recovery time, induction time, involuntary movements, and respiratory depression, might be driven by the esketamine at a dose of 0.25 mg/kg.

In addition to the benefit observed in the overall analysis of low-dose esketamine, it is worth noting that esketamine 0.25 mg/kg significantly increased mean oxygen saturation and decreased the incidence of hypoxia. This may be because the effect of esketamine in counteracting the respiratory depression of propofol increased progressively with increasing dose and reached a statistical difference at 0.25 mg/kg. This suggests that 0.25 mg/kg may be the optimal low-dose for anesthesia in gastrointestinal endoscopy. Although this may increase the risk for hypertension, aggressive preoperative blood pressure control and circumvention in patients with poor blood pressure control can effectively reduce this risk.

### 4.5 Limitations and prospects

Although the present study adhered to the PRISMA guidelines, there were some limitations. First, this meta-analysis included only 9 studies and 1144 subjects, which may have reduced precision of the results. Second, 6 of the included studies did not report allocation concealment methods, and 3 did not describe intervention blinding, which increased the potential risk for selection and implementation bias. Third, the experimental centers of all included studies were located in China, which means that the studies mainly revealed the effects of low-dose esketamine on Chinese adults. Fourth, the FDA has not yet approved esketamine for anesthesia, and other countries have not yet conducted the relevant clinical trials. Therefore, the role of esketamine in painless gastrointestinal endoscopy in other racial populations remains unclear.

We anticipate that future studies will be improved in the following aspects. First, multinational, large-sample, stratified study protocols will be designed to further explore the effects of different examination modalities, age, gender, weight, blood pressure, cognitive level, and other factors on the benefits and risks of esketamine in painless gastrointestinal endoscopy. Second, high-quality clinical studies should aim to explore the effects of low-dose esketamine on different outcomes of painless gastrointestinal endoscopy in adults, and to provide more supportive data for evidence-based research. Third, design clinical trials to compare the risks and benefits of different dosages of esketamine in adults undergoing painless gastrointestinal endoscopy, and to determine the optimal dose of esketamine to assist propofol anesthesia.

## 5 Conclusion

Low-dose esketamine was safe and effective for painless gastrointestinal endoscopy, and 0.25 mg/kg appeared to be the optimal dose in the dosage ranges examined. However, caution should be exercised when administering this drug to patients with inadequate preoperative blood pressure control.

## Data Availability

The original contributions presented in the study are included in the article/Supplementary Material, further inquiries can be directed to the corresponding author.
